# Barriers and Facilitators to the implementation and scale-up of a mHealth integrated care program for diabetes and hypertension in Ghana: a qualitative study of the Akoma Pa program

**DOI:** 10.1186/s12913-026-14175-0

**Published:** 2026-02-21

**Authors:** Luise Struss, Paulina Afia Gyinae Wilberforce, Daniel Opoku, Daniel Boateng, Kofi Akohene Mensah, Anthony Kwaku Edusei, Cornelia Henschke, Wilm Quentin, Verena Struckmann

**Affiliations:** 1 Berlin School of Public Health, Charité – Universitätsmedizin Berlin, Berlin, Germany; 2 Department of Health Policy, Management, and Economics, School of Public Health, Kwame Nkrumah University of Science and Technology, Kumasi, Ghana; 3 Department of Health Care Management, Technical University, Berlin, Germany; 4 German West-African Centre for Global Health and Pandemic Prevention, Berlin, Germany; 5 Department of Epidemiology and Biostatistics at the School of Public Health, Kwame Nkrumah University and Technology, Kumasi, Ghana; 6 Department of Health Promotion and Disability Studies, School of Public Health, Kwame Nkrumah University of Science and Technology, Kumasi, Ghana; 7 Institute of General Practice and Interprofessional Care, University Hospital Tübingen and Faculty of Medicine, Eberhard Karls Universität Tübingen, Tübingen, Germany; 8 Chair of Planetary & Public Health, Universität Bayreuth, Bayreuth, Germany

**Keywords:** Digital health technologies, Mobile health, Non-communicable diseases, Tele-counselling, Ghana

## Abstract

**Objective:**

To determine the barriers and facilitators to the implementation and scale-up of the Akoma Pa mHealth-based integrated care program for diabetes and hypertension in Ghana.

**Methods:**

30 implementation managers and healthcare providers (Akoma Pa champions) were interviewed in-depth based on their experience and ability to provide information on implementing and scaling-up the Akoma Pa program in February 2024. Participants were selected from the Christian Health Association Ghana healthcare facilities across Ghana. Thematic qualitative content analysis based on Kuckartz was used to identify recurring themes and categories, guided by the mHealth Predisposing Characteristics, Needs, and Enabling Resources (PNE) Framework.

**Results:**

Participants identified 47 factors influencing the implementation and scale-up of the intervention, categorized into provider- and patient-level contextual factors. Findings were organized according to perceived usefulness and perceived ease of use, reflecting assessments of value, feasibility, and scale-up potential. Key enablers included mobile phone accessibility, provider engagement, and integration with the National Health Insurance Scheme. Reported barriers included digital infrastructure gaps, policy misalignment, lack of data protection policies and workforce limitations. Although the SPICE app, central to the Akoma Pa digital health program, enhanced patient tracking and care coordination, it still required ongoing support to ensure interoperability and scalability. Evidence-based practices and personalized care improved patient management and outcomes.

**Conclusion:**

Integrated mHealth programs such as Akoma Pa hold promise for strengthening chronic disease management in low-resource settings. Addressing infrastructural and long-term funding barriers is critical to sustaining and scaling these interventions. Findings offer insights to inform future digital health policy and program design in sub-Saharan Africa.

**Supplementary Information:**

The online version contains supplementary material available at 10.1186/s12913-026-14175-0.

## Introduction

Noncommunicable diseases (NCDs), such as hypertension, diabetes, and cancer, caused an estimated 41 million deaths in 2021, and this keeps increasing [1]. Cardiovascular diseases (CVDs) are the leading contributors, responsible for 17.9 million deaths, while diabetes accounts for about 2 million annually. Hypertension and diabetes are major, modifiable risk factors for CVD and premature NCD-related deaths [1, 2].

NCDs are projected to cause 77.6% of global disability-adjusted life years (DALYs) by 2050, a 13.4% rise from 2022 [3]. This burden is rapidly increasing in sub-Saharan Africa (SSA), where CVDs account for 13% of all deaths and 37% of NCD-related mortality [4]. Underfunded health systems, limited infrastructure, and healthcare workforce shortages exacerbate challenges in managing chronic diseases [5–7]. Inadequate screening, low awareness, and limited access to diagnostic services and trained providers, in particular in rural areas, further hinder early detection and care intervention [5–8].

In Ghana, NCDs now account for approximately 45% of all mortalities, with CVD, cancer, chronic respiratory conditions, and diabetes as the predominant contributors [9]. The disease burden is expected to rise further, driven by demographic shifts, urbanization, and behavioral risk factors such as poor diet, physical inactivity, tobacco use, and harmful alcohol consumption [10]. The national prevalence of diabetes ranges from 2.8% to 4.0%, with higher rates reported among women and in urban areas [10]. Hypertension affects roughly 30.3% of the adult population, with marked variations by age, sex, and region [11]. Alarmingly, a recent cross-sectional study found that one-third of individuals with hypertension were unaware of their condition [12]. To address this, Ghana’s Ministry of Health has implemented a national policy on NCD prevention and control, prioritizing health system strengthening, research, and sustainable financing [13]. However, operationalizing these strategies remains challenging.

The COVID-19 pandemic underscored the transformative potential of digital health technologies (DHTs) - including telemedicine and mobile health (mHealth) tools - for delivering accessible, scalable care in resource-limited settings [14, 15]. DHTs offer platforms for improved access, continuity of care, and data-driven decision-making [16]. In SSA, mobile phone penetration offers a promising opportunity for mHealth integration. Evidence suggests that mHealth interventions can enhance chronic disease care, promote adherence, and improve outcomes cost-effectively [17–21]. In Ghana, studies such as Opoku et al. have shown that mHealth solutions can support care delivery through patient tracking and real-time data utilization [20]. Yet, broader implementation remains limited by poor system interoperability, policy gaps, and infrastructural constraints [21]. Understanding the contextual factors that influence mHealth scale-up is critical to realize their full potential.

To address these challenges, Medtronic Labs, the Christian Health Association of Ghana (CHAG), the Deutsche Gesellschaft für Internationale Zusammenarbeit (GIZ), and Novartis launched the ‘Akoma Pa’ (meaning healthy or good heart) program. This mHealth-based integrated care model is designed to enhance the diagnosis, treatment, and long-term management of diabetes and hypertension in Ghana [22].

The program integrates digital health tools, personalized care plans, and coordinated service delivery to streamline chronic disease management. Initially implemented across 85 public health facilities in 8 out of 16 regions in Ghana, Akoma Pa’s pilot phase (December 2021–February 2023) provided all medical supplies through program funding. A subsequent sustainability phase, launched in April 2023, incorporated the program into Ghana’s National Health Insurance Scheme (NHIS).

At the core of Akoma Pa is the SPICE digital app, developed by Medtronic Labs. SPICE facilitates community-based screening, real-time patient monitoring, personalized care planning, and data analytics. Designed for both community health workers and clinical staff, SPICE fosters patient engagement and enables data-informed decision-making.

Despite growing interest in DHTs, there is limited research on the system-level, organizational, and individual factors influencing implementation and scale-up of integrated mHealth programs in SSA. This study aims to address that gap by examining the implementation and scale-up of the Akoma Pa program in Ghana from the perspective of implementation managers and healthcare providers (Akoma Pa champions). The analysis is guided by the Predisposing Characteristics, Needs, and Enabling Factors (PNE) framework developed by Opoku et al. [23], which is particularly suited for capturing the complex contextual dynamics of low-resource health systems. Insights from this study will inform future policy and practice to optimize integrated mHealth interventions for chronic disease management in Ghana and comparable settings.

## Methods

### Study design

For this exploratory study, a qualitative research design was applied using in-depth interviews (IDIs) to collect data. The study is part of a larger research program that uses quantitative and qualitative methods to evaluate the implementation process and potential scale-up of a mHealth intervention in Ghana. Given that participants were drawn from a defined network of Akoma Pa Champions and could therefore be potentially identifiable within professional circles, particular care was taken to ensure confidentiality. While complete anonymity could not be absolutely guaranteed due to the small and specialized participant pool, all efforts were made to minimize the risk of identification in data handling, analysis, and reporting. The ethical approval for the study was granted by the Christian Health Association of Ghana (CHAG) Institutional Review Board (IRB) (reference number: CHAG-IRB02072023; 17th of January 2024) and the Kwame Nkrumah University of Science and Technology (KNUST) Committee on Human Research, Publications, and Ethics (reference number: CHRPE/AP/220/24; 2nd of April 2024).

### Data collection tool and analysis

Data collection was conducted in February 2024. Although 46 CHAG-affiliated facilities implementing the Akoma Pa program were initially approached, in-depth interviews (IDIs) were ultimately completed with representatives from 30 facilities. To facilitate participation, interviews were conducted at three designated sites in Ghana—two in Kumasi and one in Mankessim—based on participants’ proximity. In total, 30 respondents participated in IDIs guided by a semi-structured interview guide developed specifically for this study (Supplemental Material 1).

The guide was constructed by PW, drawing on the study’s objectives and the mHealth PNE framework developed by Opoku et al. [20] The draft instrument underwent multiple rounds of expert review by DO and VS to ensure content validity.

Pilot testing was conducted at the Church of God Clinic in Essienimpong, a CHAG affiliated facility that serves Akoma Pa patients but was not among the 46 facilities sampled for this study [24]. The pilot interview with two Akoma Pa champions aimed to assess the guide’s reliability, validity, logical flow, clarity, and cultural sensitivity. Based on the pilot findings, revisions were made prior to the main data collection.

All interviews were conducted by PW, with the support of a trained research assistant. Written informed consent was obtained from each participant prior to the interview (Supplemental Material 2). Interviews were audio recorded with permission and complementary field notes were taken to capture off-tape observations. A total of 30 IDIs was conducted, each lasting approximately 35 min. Interviews continued until a saturation point was achieved and no new themes emerged in the final three interviews.

Audio recordings were transcribed verbatim. The transcription of the audio recordings was conducted by PW and a trained research assistant with the support of AI (turboscribe a.i.) for 10 transcripts, the remaining 20 audio recordings were transcribed manually. The transcripts were then cross-checked again with the audio recordings to ensure accuracy. Thematic analysis followed Kuckartz’s structured method for qualitative content analysis using the main categories from the PNE Framework as well as inductively developed categories evolving in the coding process as subcategories and was facilitated using MAXQDA 2024 (VERBI Software) [25, 26]. To ensure intercoder reliability, LS and PWindependently coded the first five transcripts. This dual-coding process was extended to five additional transcripts to refine the number and naming of the subcategories, as the coders had initially generated slightly different category sets. LS then completed coding of the remaining 20 transcripts.

**Fig. 1:**
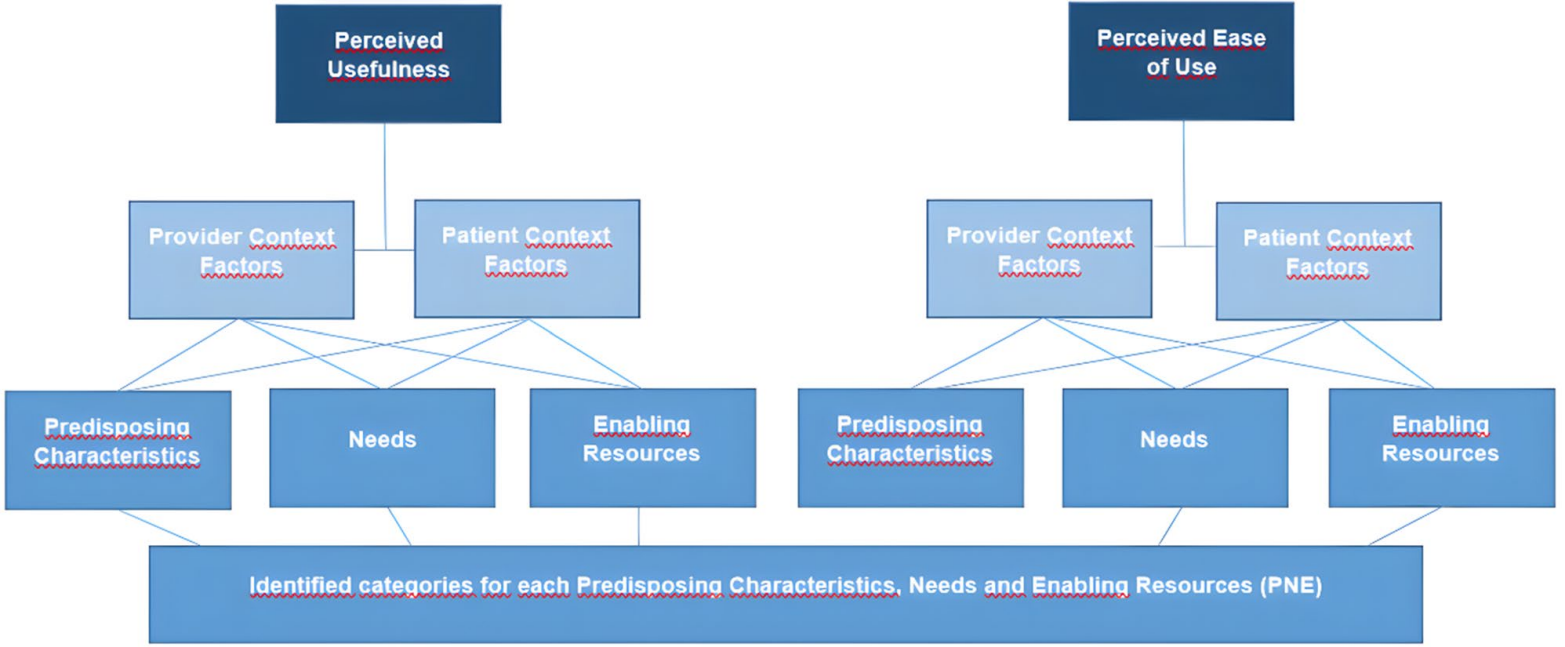
Coding frame of the analysis using the mHealth Predisposing Characteristics, Needs and Enabling Resources Framework (PNE) based on Opoku et al. 2019 [20]

The coding structure was collaboratively reviewed and refined by LS, PW, DO, and VS. A mixed deductive–inductive analytical approach was employed. Deductively, the analysis was guided by the PNE framework [20], which conceptualizes how contextual factors—Predisposing Characteristics, Needs, and Enabling Resources—influence both Perceived Usefulness and Perceived Ease of Use among patients and providers (Fig. 1; Table 2). Inductively, emergent categories were added based on the data to supplement the framework.

The PNE framework was used to examine both healthcare provider and patient context factors that shape perceptions of the Akoma Pa intervention’s perceived usefulness and perceived ease of use. These are critical components for assessing the intervention’s implementation success and scalability.

For healthcare providers, the analysis considered:

- **Predisposing Characteristics** (e.g., staff training, supervision, and commitment),
- **Needs** (e.g., workload demands, resource deficits),
- **Enabling Resources** (e.g., physical and technical infrastructure, coordinated services, and integrated care delivery) [20].

Although patients were not interviewed directly, the analysis explored patient-related factors as reported by healthcare providers, including:

- **Predisposing Characteristics** (e.g., rural location, health literacy, educational background),
- **Needs** (e.g., barriers to access, financial burden of healthcare),
- **Enabling Resources** (e.g., service affordability, accessibility, person-centered care).

### Participants

Study participants were purposely selected from among the Akoma Pa “Champions”—members of the Christian Health Association of Ghana (CHAG) who served as both facility-level implementers and healthcare providers for the Akoma Pa program (Table 1). Written invitations describing the study’s purpose and objectives were distributed to Akoma Pa Champions at selected CHAG facilities via Medtronic Labs Ghana and CHAG.

**Table 1:** Participants` main characteristics

Champions	(*N* = 28) *
**Age**, (in years) mean (range)	34 (26–41) **
**Gender**	
Male	15
Female	13
**Years** working for the intervention, mean (range)	2 (1,5 − 3) **

*Information about demographic characteristics was available for 28 out of 30 champions

**Decimal numbers were rounded to whole numbers

**Fig. 2:**
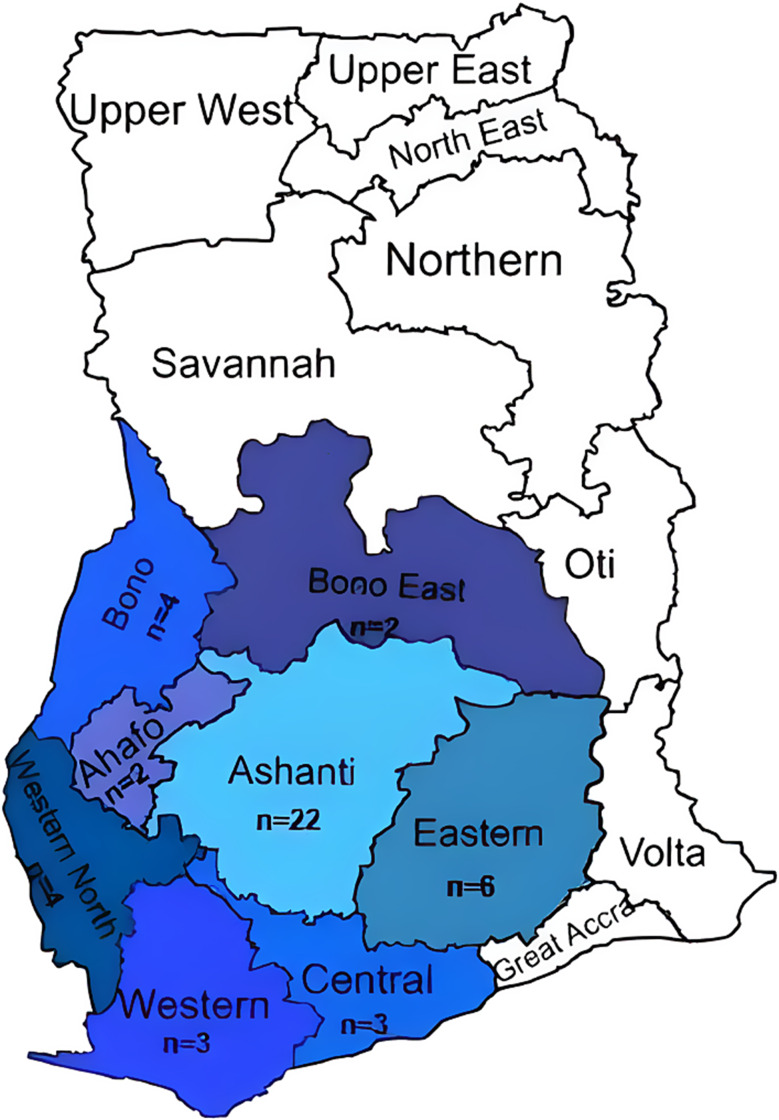
Map of Ghana highlighting the eight regions and the number of facilities (n) selected for the study pertaining to the Akoma Pa intervention, 46 facilities in total

Of the 46 Champions contacted from 46 facilities, 30 (65%) responded, met the eligibility criteria, and consented to participate in the study (Fig. 2).

Eligibility required current engagement as a CHAG-designated Champion in the Akoma Pa intervention. Participation was voluntary and contingent on availability during the data collection period; no additional exclusion criteria were applied. Participants received lunch and a modest transportation allowance as compensation for their travelling to the interview sites.

## Results

In this study, perceived usefulness and perceived ease of use were treated as key implementation outcome indicators, reflecting participants’ assessments of the intervention’s acceptability, practicality, and potential for scale-up. A total of 30 in-depth interviews with Akoma Pa Champions were conducted. According to Medtronic Labs, the implementation partner of Akoma Pa, 85 CHAG health facilities in eight regions across Ghana have implemented the intervention and as such were considered as the study population (Fig. 2). Table 1 summarizes the demographic characteristics of the 28 out of 30 study participants who provided information. Thirteen (13) of the participants were female, with a mean age of 34 years (see Table 1). On average, the champions had worked with the Akoma Pa program for two years. In addition to their roles as Akoma Pa facility managers, all participants were practicing medical professionals including nurses, medical doctors, physician assistants, medical laboratory scientists, pharmacists, anesthetists, and health information officers.

Overall, 47 factors influencing the implementation and scale-up of the intervention were identified (see Table 2).

**Table 2:** Summary of identified predisposing characteristics, needs and enabling resources for the implementation and scale-up of an mHealth based integrated care intervention

Perceived Usefulness (*n*)* ^frequency of mentions^			Perceived Ease of Use (*n*)*	
	Provider Context Factors	Patient Context Factors	Provider Context Factors	Patient Context Factors
Enabling Resources	• ICT-Infrastructure (42) • Local Ownership & Integration in NGO-/ National Policy (13) • Financial Partnerships & Piloting Support (24) • Available Physical and Technical Resources (120) • Evidence Based (11) • Coordinated Services & Integration of Care (53) • Management Support & Commitment (17)	• Adequate infrastructure & Resources (8) • Person Centeredness (20) • Convenience, Affordability & Availability of Services (93)	• Data Management & Access (39) • User-Friendly Interface (32) • Maintenance & Technical Support (40) • Extensive Interface & Interoperability (18)	• Family & Community Support (2) • Health Insurance (6) • Means of Identification (6)
Needs	• Service Delivery (48) • Fostering Expectations (22) • Misuse and Misappropriation of Resources (3) • Transparency of Data Protection (18) • Resource gaps • Medical Supply (88) • Human Resources (31) • Financial incentives (140) • Workload Burden (71) • Inappropriate Workplace Behavior (1)	• Management of Defaulters (32) • Awareness Raising (18) • Improved Healthcare & Reduction of Disease burden (65) • Polypharmacy Challenges (11) • Information Accuracy (16) • Financial Burden of Medical Costs (41) • Access Barriers (14)	• Effective Digital Tools & Features (27)	• Waiting Time (11) • Tele-Counselling Support (9)
Predisposing Characteristics	• Staff Engagement & Commitment (74) • Good Patient-Provider Relationship (11) • Attitude & Willingness (37)	• Health Literacy & Education (30) • Locality (Rural) (13) • Attitude (39) • Patient Commitment & Attendance (9)	• Digital Literacy & Education (22) • Continuous Training, Monitoring & Supervision (94)	• Continuous Technological Access Barriers (3)

Most barriers and facilitators affecting the implementation and scale-up of the Akoma Pa intervention were identified at both the provider and patient levels. Key challenges were related to resource availability, ICT infrastructure, provider workload, service affordability, and patient accessibility.

Despite these constraints, both providers and patients reported positive experiences with the intervention and perceived improvements in clinical outcomes, including reduced treatment defaulting, increased patient health literacy, and better management of chronic conditions. While some providers experienced an increased workload, patients reported shorter waiting times, suggesting a redistribution rather than a simple increase in system burden.

Financial and material incentives, together with geographical location, emerged as important cross-cutting factors influencing the success of implementation and scale-up.

Detailed participant quotes for the corresponding categories can be found in supplementary material 3.

To clarify the analytical structure, findings are presented according to the two key implementation outcome indicators; ‘perceived usefulness’ and ‘perceived ease of use’, which reflect participants’ assessments of the intervention’s value, feasibility, and scale-up potential. Within each indicator, results are organized by provider and patient context factors and further structured using the PNE framework. Barriers and facilitators are integrated within each subtheme.

### Perceived usefulness

#### Provider context factors

##### Enabling resources

Enabling resources such as reliable ICT infrastructure, adequate technical resources, and management support strongly shaped the perceptions of the SPICE application’s usefulness and scalability. Connectivity challenges, especially in rural areas, were frequently described as barriers:

*I think [… in] Ghana*,* our network is not that good of quality. I think the main challenge will be the network.* (Champion 14, Pos. 39)

To mitigate these limitations, several interviewees recommended developing an offline mode of the app, highlighting the importance of adaptable technological solutions in resource-constrained settings. Integration of care, including screening, education, treatment, and follow-up, was reported to enhance interdepartmental collaboration between consultation, laboratory, and pharmacy units. Access to adequate physical and technical resources, along with data-driven, evidence-based practices embedded in the SPICE app, further reinforced its perceived usefulness.

Financial and institutional support enabled early implementation, but long-term sustainability was viewed as dependent on national policy integration and local ownership. Concerns were raised about reliance on foreign funding.

##### Needs

Systemic shortages of staff, medical supplies, and financial incentives influenced the perceptions of usefulness. Dual documentation in both the national system and SPICE created workload burdens:

*Initially*,* what we were doing was that you see the clients on the LHIMS [Lightwave Health Information Management System]*,* which is our health information and health management system. Then*,* you see the patients on the SPICE app as well. […] So it wasn’t easy because we were spending a lot of time doing that.* (Champion 20, Pos. 38)

Uncertainty regarding data usage and program eligibility further complicated implementation. Additionally, isolated cases of misuse, such as the removal of equipment from facilities and harassment incidents were reported. Despite these barriers, providers emphasized improvements in care coordination, follow-up, and clinical outcomes.

##### Predisposing characteristics

Staff motivation, attitudes, and provider–patient relationships influenced perceived usefulness. Attitudes towards the intervention were found to range from enthusiastic to skeptical. Financial incentives initially boosted staff engagement, while positive relationships enhanced satisfaction and trust for both provider and patients:

*When you call them*,* they feel very loved. […] my healthcare provider actually thinks of me.* (Champion 20, Pos. 78)

#### Patient context factors

##### Enabling resources

It was frequently noted that patients benefited from reduced financial and logistical barriers, with the intervention facilitating easier access to medical services and reducing transportation costs. High-quality medications and person-centered care further increased patient satisfaction.

*That is when you ask anybody about Akoma Pa*,* they`ll tell you: Oh*,* Akoma Pa […] your medications are good* (Champion 21, Pos. 85)

However, limited resources and the introduction of out-of-pocket payments were highlighted as barriers for the patients to take part in the intervention.

##### Needs

The cost of medication has shown to be a key barrier during the sustainability phase, with patients frequently shifting to National Health Insurance Scheme (NHIS)-covered options. Champions emphasized the need for better awareness-raising strategies to boost patient engagement. Although the first phase offered free medications, its discontinuation led to reduced adherence.

Access barriers, polypharmacy, and medication side effects were noted as persistent challenges. Nonetheless, improved disease tracking, reduced defaulter rates, and better health outcomes were widely acknowledged:

*We were able to track our patients […] it really helped.* (Champion 6, Pos. 47)

##### Predisposing characteristics

While many patients had not been aware of their conditions prior to the intervention, the program was seen as fostering more proactive health-seeking behaviors and disease awareness. However, geographic challenges, particularly for rural residents, were a notable constraint:

*And also getting to where they are because we are from villages*,* trying to reach them was not easy. They coming to us was not easy. The distance was also a challenge. (Champion 12*,* Pos. 31)*

### Perceived ease of use

#### Provider context factors

##### Enabling resources

The SPICE app’s intuitive and user-friendly interface and availability of technical support were commonly highlighted as critical enablers of usability. However, limited data management functionalities and insufficient backend access and the demand for interoperability were identified as constraints.

A recurring recommendation was enhancing interoperability with existing national health information systems to reduce duplication of work: *If it is incorporated with the systems we are using currently*,* we wouldn’t feel it is giving us double work.* (Champion 23, Pos. 92)

##### Needs

It was commonly reported that the digital tools and features of the SPICE app were helping them carrying out the intervention.

*Akoma Pa is also a type of the e-health. And I think*,* like I said earlier on*,* Akoma Pa is good because with the app that we are using*,* there are a lot of information on it. We can even chat with the clients on the app. So*,* it’s very helpful.* (Champion 19, Pos. 18)

##### Predisposing characteristics

It was commonly noted that there were differences in digital literacy levels across providers influencing the champion’s perceptions of the ease of use. Providers’ with higher levels of digital literacy and education showed a greater ability to engage with the intervention than those with a lower digital literacy and education. Embedded within the communities they serve, Medtronic LABS’ field operations teams partnered with the communities to train workers in coordinating care activities and to refine digital tools, through data reviews, tactical planning, and innovations such as client portals and tele-calling systems, enhancing patient monitoring, data access, and overall program effectiveness [27]. Continuous training and supportive supervision were considered essential for maintaining implementation fidelity:

*We were trained to do it and […] we were pre-informed of any update so there was no challenge.* (Champion 28, Pos. 41)

#### Patient context factors

##### Enabling resources

Enabling resources such as the support from insurance, family, and community members was pointed out to facilitate patient engagement with the intervention. Health insurance was reported as an important enabler of service access and during the registration process. Family and community support was described as facilitating patient engagement, particularly in navigating technological and logistical challenges by family and community members making their phones available for the patient to participate.

*When patients do not have phone numbers*,* we use our phone number and they bring a relative’s phone number on the next visit*,* so that we can reach them later.* (Champion 29, Pos. 32)

##### Needs

Champions mentioned needs such as long waiting times to be frustrating for patients and occasionally discouraged their attendance which in the champion’s view hindered the ease of use of the intervention. However, tele-counseling has shown to be a well-accepted key facilitator, providing education and support, and reaching out to patients from various areas.

*Later on*,* the Akoma Pa program was already doing the tele-counselling. So*,* we took it upon ourselves that sometimes we call them*,* and then we inform them about their visits*,* their next visits*,* and inquire from them how they are doing in their various zones.* (Champion 10, Pos. 16)

##### Predisposing characteristics

Technological access barriers, including a lack of devices and old age, were noted to limit the intervention’s scalability for some patients and consequently lowering the perceived ease of use of the project:

*Plenty of the ages*,* they don’t use phones […] and some […] don’t use the Android phones.* (Champion 15, Pos. 61)

## Discussion

The effective implementation of mHealth interventions remains essential in addressing equity gaps in healthcare delivery, particularly in rural, remote, and resource-constrained environments [28]. This study provides valuable insights into the factors that facilitated or hindered the implementation and potential scalability of Akoma Pa, an integrated mHealth program in Ghana. Guided by the mHealth PNE framework, we conducted qualitative interviews with healthcare providers, we explored both patient and provider experiences with the intervention.

Overall, our findings demonstrate broad stakeholder acceptance of Akoma Pa, with both patients and providers highlighting its relevance, usability, and potential to enhance chronic disease management. Several key determinants of implementation emerged. These included the adequacy of information and communication technology (ICT) infrastructure, funding mechanisms, provider incentives, availability of medical resources, continuous training, and a patient-centered design. The mHealth PNE framework proved valuable in capturing the multi-level contextual enablers and barriers to implementation, offering a comprehensive lens through which to assess intervention dynamics. As Akoma Pa transitions from pilot phase to scale-up, its long-term sustainability will hinge on addressing these systemic and infrastructural challenges. Strengthening these foundational elements will be essential to improving outcomes for individuals living with diabetes and hypertension in Ghana and similar settings.

### Facilitators of implementation and scale-up

Program champions identified ongoing training, supervision, and monitoring as critical facilitators. These elements enhanced usability, increased confidence in the system, and supported consistent implementation across sites. These findings are aligned with prior studies that highlight training and capacity-building as key to sustaining and scaling mHealth interventions [29, 30].

Participants also emphasized the importance of institutionalizing Akoma Pa within national health policies. Integration into existing governance structures and alignment with national digital health strategies were seen as crucial steps toward ensuring long-term viability. This is consistent with literature underscoring the need for mHealth solutions in SSA to be embedded within national health systems, with active stakeholder engagement to ensure sustainability [29, 30].

The SPICE app, the digital/technological backbone of the intervention, was viewed positively by providers. Its clinical decision-support tools, visual dashboards, and patient tracking functions supported evidence-based care and enabled more engaging, effective interactions between providers and patients. Participants reported that patients responded well to the tele-counseling components and appreciated the personalized care the app facilitated. Additionally, perceived improvements in clinical outcomes—such as reduced defaulters, enhanced patient literacy, and better chronic disease management—were noted. These observations align with a recent meta-analysis, which found that mHealth interventions can significantly improve patient adherence, health literacy, and overall quality of life in individuals managing diabetes and hypertension [31–33].

### Barriers to implementation

Despite high acceptability, several challenges regarding the implementation and scale-up were noted. The most important among them was unreliable network connectivity, which limited access to patient data and delayed care delivery. The SPICE app’s lack of interoperability with other digital health information systems further compounded this issue, increasing workload by manual data entry burdens and impeding coordinated care. Participants recommended the development of an offline mode to mitigate internet dependency which constitutes a novel suggestion only rarely reported in prior literature [34].

Indeed, the need for greater integration and interoperability was underscored by a recent study advocating for unified digital health architectures to support integrated care pathways [35]. Champions highlighted that expanding SPICE’s usability across different user groups, including patients themselves, could further strengthen the integration of care in the program. These views are consistent with prior work emphasizing the role of robust ICT infrastructure in minimizing inefficiencies and reducing the administrative burden of mHealth interventions [36, 37].

Financial and motivational incentives also played a pivotal role. During the early phases of the program, providers received monetary compensation or other incentives, which supported their engagement. When these incentives were withdrawn, providers’ motivation reportedly declined. This supports existing findings that documented the importance of user incentives for continued mHealth engagement and workflows [30, 38–40].

Patient-level financial barriers were also salient. Transitioning from free medication access under Akoma Pa to NHIS-funded prescriptions required patients to either pay out-of-pocket (OOP) for preferred medications or switch to NHIS-covered alternatives. This shift often disrupted continuity of care, with potential consequences such as adverse drug reactions or poorer glycemic and blood pressure control. These concerns are consistent with studies linking abrupt medication changes to increased cardiovascular risk and therapy discontinuation [41, 42]. Ensuring that NHIS formularies include the high-quality medications used in Akoma Pa could support continuity, improve adherence, and reduce inequities in access.

Other resource limitations, particularly inconsistent availability of glucose test strips, further constrained effective care delivery. Similar supply chain issues have been reported in prior studies, where delays in NHIS reimbursements contributed to stock-outs and interrupted care [7]. Addressing these systemic challenges within NHIS operations could greatly enhance the scalability and performance of integrated mHealth programs.

### Methodological strengths and limitations

A major strength of this study was the use of the mHealth PNE framework, which facilitated a structured, multi-level analysis of implementation drivers. Complementary use of the Kuckartz method allowed for a more nuanced integration of inductive themes within a deductive analytic structure. The study’s embedding within a broader research initiative will also enable future quantification of the identified factors across a larger, more representative sample.

Nevertheless, the study had limitations. Patient perspectives were reported second-hand through program champions, potentially introducing reporting bias. Moreover, as the interviews required participants to recall events over a year in the past, recall bias may have influenced responses. Direct engagement with patients in future research would provide a more complete understanding of the intervention’s impact. Additionally, the findings are context-specific and may not be generalizable across regions or mHealth programs.

The study did not assess all dimensions of scalability, such as cost-effectiveness, long-term user retention, or institutional readiness. Furthermore, applying the PNE framework offered a structured lens for analyzing provider- and patient-level determinants, but category overlap complicated classification. Factors such as ICT infrastructure, financial incentives, or health insurance support could be coded as enabling resources, needs, or predisposing characteristics, illustrating that domains are not always mutually exclusive and often operate across dimensions. Finally, the PNE framework does not address broader scalability concerns such as long-term financing, institutional readiness, or policy integration. Thus, while very useful for structuring analysis, it may require combination with complementary frameworks (e.g., CFIR, WHO building blocks) to support system-level recommendations. Participation in the study was voluntary, 30 of 46 invited Akoma Pa Champions took part in the interviews. Champions who were more engaged with the intervention, had more positive experiences, or had greater availability to travel to interview sites may have been more likely to participate. These factors introduce a risk of self-selection bias and may have resulted in a sample that reflects more motivated or better-resourced implementers. Findings should therefore be interpreted as reflecting the experiences of participating Champions and may not fully represent the views of all facilities involved in the Akoma Pa intervention.

### Implications for digital health policy and practice

This study contributes novel insights in two relatively underexplored areas: the implementation need for offline-capable digital health functionalities in low-connectivity settings, and the real-world implications of medication financing transitions in integrated care settings. Our findings emphasize the critical need to address ICT development gaps with a long-term, system-oriented approach, including continuous engagement with healthcare professionals and end users to ensure that digital systems are tailored to the specific needs of providers and patients. Such engagement is essential to avoid increasing administrative burdens and to ensure that digital tools enhance rather than hinder professional work. Beyond the issue of financing, there is broad consensus that integrated care requires new payment models. These should include pooled budgets, shared savings or loss arrangements, and mechanisms for tracking and incentivizing quality improvement [43–45]. Without such structural changes, fragmented care delivery is likely to persist.

To enhance the scalability and sustainability of mHealth programs such as Akoma Pa, future strategies should focus on strengthening digital infrastructure, aligning with existing regulatory frameworks, and ensuring financial security through rapid integration into national funding mechanisms and insurance schemes. Such efforts will be critical to unlocking the full potential of mHealth interventions in reducing health inequities and improving chronic disease management in low-resource settings.

## Conclusion

This study explored the barriers and facilitators to the implementation and scale-up of the Akoma Pa mHealth-based integrated care program in Ghana, giving crucial insights into the factors impacting its success. To strengthen the program, targeted efforts are needed to improve ICT infrastructure, particularly by ensuring stable network connectivity and enhancing interoperability of digital tools. Additionally, securing long-term financing and embedding the intervention into national health policies are essential steps to ensure its sustainability and scalability. The findings are particularly valuable for policymakers, healthcare providers, and program implementers, providing implications for addressing challenges and optimizing resource allocation. For instance, aligning medication regimens with NHIS provisions could dismantle financial barriers for patients, while consistent training and management support could improve provider engagement and efficiency. Program designers could draw on these insights to better adapt mHealth interventions to the specific needs of resource-limited settings, ensuring equitable access and sustained improvements in healthcare delivery. By addressing these identified areas, Akoma Pa and similar initiatives can contribute meaningfully to achieving universal health coverage in Ghana and beyond, serving as a replicable model for mHealth-based integrated care programs in other low- and middle-income countries.

## Supplementary Information

Below is the link to the electronic supplementary material.

Supplementary Material 1

## Data Availability

The datasets used and analysed during the current study are available from the corresponding author on reasonable request.

## References

[1] World Health Organization. Noncommunicable diseases: key facts. https://www.who.int/news-room/fact-sheets/detail/noncommunicable-diseases. Accessed 22 May 2024.

[2] Institute for Health Metrics and Evaluation. GBD Global Burden of Disease Collaborative Network, Global Burden of Disease Study 2019 (GBD 2019) Results. https://vizhub.healthdata.org/gbd-results/. Accessed 2 Jun 2024.

[3] GBD 2021 Forecasting Collaborators. Burden of disease scenarios for 204 countries and territories, 2022–2050: A forecasting analysis for the global burden of disease study 2021. Lancet. 2024;403(10440):2204–56. 10.1016/S0140-6736(24)00685-8. 38762325 10.1016/S0140-6736(24)00685-8 PMC11121021

[4] Yuyun MF, Sliwa K, Kengne AP, Mocumbi AO, Bukhman G. Cardiovascular diseases in Sub-Saharan Africa compared to high-income countries: an epidemiological perspective. Glob Heart. 2020;15(1):15. 10.5334/gh.403. 32489788 10.5334/gh.403 PMC7218780

[5] Gouda HN, Charlson F, Sorsdahl K, et al. Burden of non-communicable diseases in Sub-Saharan Africa, 1990–2017: results from the global burden of disease study 2017. Lancet Glob Health. 2019;7(10):e1375–87. 10.1016/S2214-109X(19)30374-2. 31537368 10.1016/S2214-109X(19)30374-2

[6] Sorato MM, Davari M, Kebriaeezadeh A, et al. Reasons for poor blood pressure control in Eastern Sub-Saharan africa: looking into 4P’s (primary care, professional, patient, and public health policy) for improving blood pressure control: A scoping review. BMC Cardiovasc Disord. 2021;21(1):123. 10.1186/s12872-021-01934-6. 33663387 10.1186/s12872-021-01934-6 PMC7971125

[7] Laar AK, Adler AJ, Kotoh AM, et al. Health system challenges to hypertension and related non-communicable diseases prevention and treatment: perspectives from Ghanaian stakeholders. BMC Health Serv Res. 2019;19:693. 10.1186/s12913-019-4571-6. 31615529 10.1186/s12913-019-4571-6 PMC6792211

[8] Witter S, Zou G, Diaconu K, et al. Opportunities and challenges for delivering non-communicable disease management and services in fragile and post-conflict settings: perceptions of policymakers and health providers in Sierra Leone. Confl Health. 2020;14:3. 10.1186/s13031-019-0248-3. 31921333 10.1186/s13031-019-0248-3 PMC6945746

[9] World Health Organization – Ghana. Ghana STEPS report 2023: nationwide non-communicable diseases risk factors assessment using the World Health Organization’s STEPwise approach in Ghana. Accra: World Health Organization – Ghana; 2024 Sep. Report No.: ISBN 978-9988-3–9188–1. https://cdn.who.int/media/docs/default-source/ncds/ncd-surveillance/data-reporting/ghana/steps/ghana-2023-steps-report.pdf?sfvrsn=1691a787_8&download=true. Accessed 29 Jun 2025.

[10] Kazibwe J, Gad M, Abassah-Konadu E, et al. The epidemiological and economic burden of diabetes in ghana: A scoping review to inform health technology assessment. PLOS Glob Public Health. 2024;4(3):e0001904. 10.1371/journal.pgph.0001904. 38470940 10.1371/journal.pgph.0001904 PMC10931482

[11] Atibila F, Hoor GT, Donkoh ET, Wahab AI, Kok G. Prevalence of hypertension in Ghanaian society: A systematic review, meta-analysis, and GRADE assessment. Syst Rev. 2021;10(1):220. 10.1186/s13643-021-01770-x. 34364395 10.1186/s13643-021-01770-x PMC8349493

[12] Ellahi B, Dikmen D, Seyhan-Erdoğan B, et al. Prevalence, risk factors, and self-awareness for hypertension and diabetes: Rural–urban and male–female dimensions from a cross-sectional study in Ghana. Int J Diabetes Dev Ctries. 2023;43(5):694–708. 10.1007/s13410-022-01141-9.

[13] Ministry of Health, Ghana. National policy: non-communicable diseases. 2nd ed. 2022. https://www.moh.gov.gh/wp-content/uploads/2022/05/Ghana-NCD-Policy-2022.pdf. Accessed 23 May 2024.

[14] Hyder MA, Razzak J. Telemedicine in the united states: an introduction for students and residents. J Med Internet Res. 2020;22(11):e20839. 10.2196/20839. 33215999 10.2196/20839 PMC7690251

[15] Food and Drug Administration. Digital health innovation plan. https://www.fda.gov/media/106331/download. Accessed 16 Dec 2024.

[16] Musa SM, Haruna UA, Manirambona E, et al. Paucity of health data in africa: an obstacle to digital health implementation and evidence-based practice. Public Health Rev. 2023;44:1605821. 10.3389/phrs.2023.1605821. 37705873 10.3389/phrs.2023.1605821 PMC10495562

[17] Ngabo F, Nguimfack J, Nwaigwe F, et al. Designing and implementing an innovative SMS-based alert system (RapidSMS-MCH) to monitor pregnancy and reduce maternal and child deaths in Rwanda. Pan Afr Med J. 2012;13:31. 23330022 PMC3542808

[18] Mahmud N, Rodriguez J, Nesbit J. A text message-based intervention to Bridge the healthcare communication gap in the rural developing world. Technol Health Care. 2010;18(2):137–44. 10.3233/THC-2010-0576. 20495253 10.3233/THC-2010-0576

[19] Aranda-Jan CB, Mohutsiwa-Dibe N, Loukanova S. Systematic review on what works, what does not work and why of implementation of mobile health (mHealth) projects in Africa. BMC Public Health. 2014;14:188. 10.1186/1471-2458-14-188. 24555733 10.1186/1471-2458-14-188 PMC3942265

[20] Opoku D, Busse R, Quentin W. Achieving sustainability and scale-up of mobile health noncommunicable disease interventions in sub-Saharan africa: views of policy makers in Ghana. JMIR Mhealth Uhealth. 2019;7(5):e11497. 10.2196/11497. 31066706 10.2196/11497 PMC6524449

[21] GSMA. The mobile economy West Africa. 2019. https://www.gsma.com/solutions-and-impact/connectivity-for-good/mobile-economy/wp-content/uploads/2020/03/GSMA_MobileEconomy2020_West_Africa_ENG.pdf. Accessed 23 May 2024.

[22] Medtronic Labs. Akoma Pa, healthcare for patients with diabetes and hypertension launches in Ghana. https://www.medtroniclabs.org/insights/akoma-pa-healthcare-for-patients-with-diabetes-and-hypertension-launches-in-ghana/. Accessed 23 May 2024.

[23] Opoku D, Stephani V, Quentin W. A realist review of mobile phone-based health interventions for non-communicable disease management in sub-Saharan Africa. BMC Med. 2017;15(1):24. 10.1186/s12916-017-0782-z. 28162090 10.1186/s12916-017-0782-z PMC5292812

[24] Christian Health Association in Ghana. Homepage. https://chag.org.gh. Accessed 13 Sep 2024.

[25] MAXQDA. Software for content analysis. https://www.maxqda.com/de/. Accessed 13 Sep 2024.

[26] Kuckartz U, Rädiker S. Analyzing qualitative data with MAXQDA: text, audio, and video. Springer; 2019. 10.1007/978-3-030-15671-8.

[27] Medtronic LABS. Global health, local solutions: 2021 impact report. Medtronic LABS; 2021.

[28] Ijeh SI, Arowoogun JO, Adeniyi AO. Addressing health disparities through IT: a review of initiatives and outcomes. World J Biol Pharm Health Sci. 2024;18(1). 10.30574/wjbphs.2024.18.1.0167.

[29] Dharmayat KI, Tran T, Hardy V, et al. Sustainability of ‘mHealth’ interventions in sub-Saharan africa: A stakeholder analysis of an electronic community case management project in Malawi. Malawi Med J. 2019;31(3):177–83. 10.4314/mmj.v31i3. 31839886 10.4314/mmj.v31i3.3 PMC6895377

[30] Tumuhimbise W, Theuring S, Kaggwa F, et al. Enhancing the implementation and integration of mHealth interventions in resource-limited settings: A scoping review. Implement Sci. 2024;19(1):72. 10.1186/s13012-024-01400-9. 39402567 10.1186/s13012-024-01400-9 PMC11476919

[31] Yaqian M, Wei L, Junping W, Gang C. Impact and efficacy of mobile health intervention in the management of diabetes and hypertension: A systematic review and meta-analysis. BMJ Open Diabetes Res Care. 2020;8(1):e001225. 10.1136/bmjdrc-2020-001225. 10.1136/bmjdrc-2020-001225 PMC7523197 32988849

[32] Park S, Woo H, Kim S, et al. Real-world evidence of a hospital-linked digital health app for the control of hypertension and diabetes mellitus in South korea: nationwide multicenter study. JMIR Form Res. 2023;7:e48332. 10.2196/48332. 37603401 10.2196/48332 PMC10477930

[33] de Souza Ferreira E, de Aguiar Franco F, dos Santos Lara MM, et al. The effectiveness of mobile application for monitoring diabetes mellitus and hypertension in the adult and elderly population: systematic review and meta-analysis. BMC Health Serv Res. 2023;23:855. 10.1186/s12913-023-09879-6. 37573312 10.1186/s12913-023-09879-6 PMC10423411

[34] Callaway DW, Peabody CR, Hoffman A, et al. Disaster mobile health technology: lessons from Haiti. Prehosp Disaster Med. 2012;27(2):148–52. 10.1017/S1049023X12000441. 22588429 10.1017/S1049023X12000441

[35] Li E, Lounsbury O, Clarke J, et al. Patient and caregiver perceptions of electronic health records interoperability in the NHS and its impact on care quality: A focus group study. BMC Med Inf Decis Mak. 2024;24:370. 10.1186/s12911-024-02789-5. 10.1186/s12911-024-02789-5 PMC11616290 39627780

[36] World Health Organization. Digital implementation investment guide: integrating digital interventions into health programmes. Geneva: World Health Organization. 2020. https://www.who.int/publications/i/item/9789240010567. Accessed 13 Dec 2024.

[37] Feldacker C, Usiri J, Kiruthu-Kamamia C, et al. Crossing the digital divide: the workload of manual data entry and integration between mobile health applications and eHealth infrastructure. Oxf Open Digit Health. 2024;2(Suppl2):ii9–17. 10.1093/oodh/oqae025. 40191682 10.1093/oodh/oqae025 PMC11936321

[38] Agbeyangi AO, Lukose JM. Telemedicine adoption and prospects in Sub-Sahara africa: A systematic review with a focus on South Africa, Kenya, and Nigeria. Healthcare. 2025;13(7):762. 10.3390/healthcare13070762. 40218059 10.3390/healthcare13070762 PMC11989057

[39] Pinter KA, Zhang H, Liu C, et al. Elements and performance indicators of integrated healthcare programmes on chronic diseases in six countries in the Asia-Pacific region: A scoping review. Int J Integr Care. 2021;21(1):3. 10.5334/ijic.5439. 33613135 10.5334/ijic.5439 PMC7879996

[40] van Kessel R, Srivastava D, Kyriopoulos I, et al. Digital health reimbursement strategies of 8 European countries and israel: scoping review and policy mapping. JMIR Mhealth Uhealth. 2023;11:e49003. 10.2196/49003. 37773610 10.2196/49003 PMC10576236

[41] Douros A, Dell’Aniello S, Yu OHY, et al. Sulfonylureas as second line drugs in type 2 diabetes and the risk of cardiovascular and hypoglycaemic events: Population-based cohort study. BMJ. 2018;362:k2693. 10.1136/bmj.k2693. 30021781 10.1136/bmj.k2693 PMC6050517

[42] Degli Esposti L, Sangiorgi D, Buda S, et al. Therapy discontinuation or substitution in patients with cardiovascular disease, switching among different products of the same off-patent active substance: A ‘real-world’ retrospective cohort study. BMJ Open. 2016;6(11):e012003. 10.1136/bmjopen-2016-012003. 27807083 10.1136/bmjopen-2016-012003 PMC5129038

[43] Struckmann V, et al. How to strengthen financing mechanisms to promote care for people with Multimorbidity in Europe? Copenhagen: WHO Regional Office for Europe; 2017. (Policy brief 24). 29144696

[44] Stokes J, et al. Towards incentivising integration: A typology of payments for integrated care. Health Policy. 2018;122(9):963–9. 10.1016/j.healthpol.2018.07.003. 30033204 10.1016/j.healthpol.2018.07.003

[45] Struckmann V, et al. How to implement integrated care? A framework with 12 overall strategies to transform care delivery. Policy brief 62. Print ISSN 1997–8065. Online ISSN 1997–8073. European Observatory on Health Systems and Policies; 2024. p. 1–34. 39836795

