# Supplementary material for: Barriers and Facilitators to the implementation and scale-up of a mHealth integrated care program for diabetes and hypertension in Ghana: a qualitative study of the Akoma Pa program
Source: BMC Health Serv Res. 2026 Feb 21;26:304. doi: 10.1186/s12913-026-14175-0 (PMC12934018; doi:10.1186/s12913-026-14175-0)
Supplement: Supplementary file 1 — Supplementary Material 1 [file 12913_2026_14175_MOESM1_ESM.docx]

# **Supplemental Material**

Supplemental Material one and two were developed by Paulina Afia Gyinae Wilberforce.

## ***Supplemental Material 1***

**SEMI-STRUCTURED INTERVIEW GUIDE FOR CHAG OFFICERS (AKOMA PA CHAMPIONS)**

**KWAME NKRUMAH UNIVERSITY OF SCIENCE AND TECHNOLOGY (KNUST) COLLEGE OF HEALTH SCIENCES SCHOOL OF PUBLIC HEALTH**

**Research Title**: Effectiveness of an Mhealth-Based Integrated Care Program for Diabetes and Hypertension Care in Ghana and Strategies for Scale-Up

**Purpose of the study:** This study aims to ascertain the barriers and facilitators of the implementation and scale-up of the Akoma Pa project test the validity and identify the main predictive factors of the PNE framework factors in determining the use and scale-up of the Akoma Pa MHealth-based integrated care program in Ghana. The PNE framework developed by Opoku et al outlines the facilitating and inhibiting factors for the adoption, sustainability and scale-up of mobile health interventions for NCDs. The framework hypothesizes that “predisposing characteristics and needs of patients and healthcare providers as well as the availability of enabling resources in the community influences the perception of patients and providers that mHealth interventions are useful and easy to use. A perception essential for the successful implementation of a mHealth intervention” (Opoku et al. 2017 and 2019).

**Name(s) and affiliation(s) of the researcher(s):** This study is being conducted by eHRPG of TU Berlin and the School of Public Health, KNUST. Researchers are Paulina Nyamekye (PhD candidate) (KNUST), Dr Verena Struckmann (TU Berlin), Dr Daniel Opoku (KNUST/TU Berlin), Dr Daniel Boateng (KNUST), Dr Aliyu Mohammed (KNUST) and Prof Anthony Edusei (KNUST).

**Part A: Demographic factors:**

Name:

Age: …

Sex:

Male

Female

**Part B: Professional Background**

Occupation/Profession…

Current place of work/facility…

Current position…

Years of work experience …………….

Your role and main responsibilities in this program...

How long have you been working on the Akoma Pa project?

**Part C: The Problem and Intervention**

1. How would you describe the Akoma PA program?
2. How have you been involved in the Akoma Pa program? /What is your involvement in the Akoma Pa program?

**Probe:** Have you been involved in the program from the start/were you one of its initiators?

1. What would you say has been your experience with the Akoma PA program?
2. What form of ICT infrastructure do you use?

**PNE FRAMEWORK**

1. Can you describe the Akoma Pa intervention? (if Part C1 not properly answered)
2. I am particularly interested in how the Akoma Pa project came to be. What did the implementation procedure look like? / What steps did you go through in the implementation?

**Probe:** What were the factors you considered before using this intervention?

- Predisposing factors (Education, Training, Attitude, Content, etc.) (What worked? What did not work and why?)
- Enabling factors (Device features, operability, suitability, policy, workload, maintenance support, etc.) (What worked? What did not work and why?)
- Need factors (Human resources capacity, Information accuracy, Characteristics of disease, etc.) (What worked? What did not work?)
- Usefulness (Disease Diagnostics, Treatment, Reduced workload, etc.) (What worked? What did not work?)
- Easy-to-use factors (Device features, Operability, Mobility, Accessibility, etc.) (What worked? What did not work?)

1. Based on your experience, what are the challenges you faced when you were trying to use Akoma Pa. How were they handled?

- Financial
- ICT Infrastructure
- Management commitment
- Staff acceptance

1. In your opinion, what are the things that are been done in the Akoma Pa program that you think is contributing to the success of Akoma Pa

**Probe:** What are the things you do that is contributing to the success of Akoma Pa

1. What are the main challenges and problems in using Akoma Pa?

**Probe:**

1. What are the things that have been done in the Akoma Pa program that you think is not contributing to the success of Akoma Pa?
2. What is the sustainability risk you envisage with this intervention?
3. Considering the idea of scaling up this intervention, what do you think will be some of the challenges this program will face in scaling up.
4. Based on your experience, what do you think will be some of the factors that can help scale up Akoma Pa?

- Financial
- ICT Infrastructure
- Management commitment
- Staff acceptance

1. May I know if there are payment systems to access Akoma Pa services?
   1. Do you know if patients have to pay for all or part of the service, they get using Akoma Pa? Yes/No …
   2. Nothing at all?
   3. If yes, what component(s) of the service delivery do they pay for and how much?
2. In what way does Akoma Pa compensate healthcare providers?
3. Who are the funders of Akoma Pa?
4. Is it supporting the integration of care?

**ISAT**

1. What were your expectations for the Akoma Pa intervention in terms of healthcare delivery for patients/ what were your expectations in terms of your work output with the introduction of Akoma Pa?
2. Were your expectations met? How?
3. Would you say that the expectations of hypertension and diabetes patients were met through Akoma Pa?
4. In your view, how did the Akoma project affect your work delivery at the clinic.
5. Would you say that the health workers have generally embraced the Akoma Pa intervention and why?
6. What have been the reported benefits/challenges in the use of Akoma Pa by patients?
7. What have been the benefits/challenges in the use of Akoma Pa by health workers?
8. Since your use of the Akoma Pa intervention, what would you say are some known unintended consequences (positive/negative) and /or adverse effects of the intervention?
9. How would you compare services given to diabetes and hypertension patients under Akoma Pa with the previous way of rendering services for diabetes and hypertension patients.
10. In addition to the issues discussed here, what other recommendations do you consider important for the improvement of the Akoma Pa program?

## ***Supplemental Material 2***

**CONSENT FORM QUALITATIVE (HEALTHCARE PROVIDERS AND IMPLEMENTORS)**

**STUDY TITLE**: Effectiveness of an MHealth-Based Integrated Care Program for Diabetes and Hypertension Care in Ghana and Strategies for Scale-Up

**Principle Investigator**:

Name: Paulina Afia Gyinae Wilberforce

Address: School of Public Health, Kwame Nkrumah University of Science and Technology Kumasi, Ghana.

Phone: 0243503326

**General Information about Research**:

In Ghana, the burden and mortality from cardiovascular diseases, particularly Hypertension and Diabetes, have achieved epidemic levels (Yawson et al., 2016). Indeed, nearly two-thirds (65%) of mortalities in Ghana are associated with non-communicable diseases (NCDs) currently (Yawson et al., 2016). Following technological advancements, mHealth has become an increasingly popular and indispensable strategy in the management of diabetes and hypertension in especially resource-constrained settings (Mao et al., 2020). In Ghana, only three mHealth interventions (the ComHIP project, Medtronic’s Labs’ Empower, and Akoma Pa program) targeted NCDs, while in effect, currently, Akoma Pa is the only mHealth intervention implemented and scaled up targeting the treatment, control, and management of NCDs (specifically Hypertension and Diabetes) (Medtronic LABS.,2022). Akoma Pa (meaning healthy or good heart) was implemented to decrease the rise of Hypertension and Diabetes in Ghana, and as a cost-effective strategy to prevent, detect, and manage these conditions. The effectiveness of most mHealth interventions for improving the management and control of diabetes and hypertension has not been ascertained in many countries (Mao et al., 2020). In Ghana, the evidence of the effectiveness of the Akoma Pa mHealth intervention in the management and control of Diabetes and Hypertension is limited. This study seeks to establish the effectiveness of Akoma Pa in the management and control of hypertension and diabetes to bridge the literature gap and to provide evidence for scale-up.

**Purpose of the study:**

This study is conducted to provide evidence of the effectiveness of the Akoma Pa mHealth program in the management of hypertension and diabetes in Ghana and to also provide evidence for factors determining the use and scale-up of the Akoma Pa mHealth-based integrated care program in Ghana.

**The procedure of the research, what shall be required of each participant, and duration:**

You will be interviewed on your perception and experience in the implementation and scale-up of the Akoma Pa mHealth-based integrated healthcare program, as well as your demographic information. This interview will be recorded for further analysis. You may also be invited to participate in filling a survey data or focus group discussion. This interview will take a maximum of 60 minutes to be completed.

**Possible Risk(s) and Discomforts: We do not expect that you will incur any risk by participating in this study** beyond the time spent in the interview/discussion. You may feel tired or experience emotional distress when talking about personal experiences. You can skip any questions you do not wish to answer. Counselling support will be available if required. **Possible Benefit(s):**

The findings will help bridge the literature gap on the effectiveness of Akoma Pa and develop recommendations for governance approaches tailored to Ghana's healthcare context and priorities in terms of mHealth. Your participation will inform efforts to advance the sustainability and scale-up of digital health interventions in Ghana for the benefit of patients, providers, and the health system. The results can catalyse policy reforms that expand access to high-quality, digitally enabled care nationwide.

**Confidentiality**:

To help you make your decision, please read this information sheet. You are free to discuss the contents of this document with someone you trust or your doctor. No identifying information like your name or address will be used for any purposes unless you consent to provide contact info for further follow-up. All data will be stored securely in our computers. All data will only be accessible to authorized study staff and handled securely and confidentially. Findings will be reported at an aggregated level without revealing individual participant details.

**Costs/Compensation:**

You will not be paid for taking part in the study. However, refreshments may be provided during interviews/group discussions. Transport reimbursement may also be available if you must travel specifically for this research to show our appreciation for your participation.

**Voluntary participation and right to leave the research:**

Participation in this study is voluntary. You have the right to decline participation or withdraw consent at any time without any penalties. You can also decline to answer any interview question you are not comfortable answering. When you agree to participate in the study, you will complete an informed consent form. A copy of the Information sheet will be given to you after it has been signed or thumb-printed to take home. Please tick ONE of the following below: I give my permission for the interview to be recorded. I do not give my permission for the interview to be recorded.

**Please read the following options below carefully and tick ONE:**

- I agree that material from my interview may be quoted and that these quotations may be attributed to me.
- I agree that material from my interview may be quoted, but I would like my name to be anonymous.
- I agree that material from my interview may be quoted, but I would like my name to be anonymized as well as any other information that might be used to identify me, including the organization that employs me and my position within it.
- I do not agree that any material from my interview may be quoted, but the researchers may use information from my interview to inform their analysis.

**Alternatives to participation:**

You may choose not to take part in this study. Your decision to participate or not will not affect your employment status or current job position.

**Consequence of withdrawal:**

There will be no negative consequences if you decide to withdraw from the study. Please note that data collected up to the point of withdrawal may still be used in analysis, as it will be anonymized without personal identifiers. We will make every effort to remove your data if you do wish to withdraw, as long as analyses have not already been performed. Your choices will be respected throughout your participation in the study.

**Contacts for additional information:**

If you have any questions, please contact the Principal Investigator, Paulina Afia Gyinae Wilberforce via mobile at +233 (0) 243503326 or through email at [panyamekye@gmail.com](mailto:panyamekye@gmail.com)

**Your rights as a participant**

This research has been reviewed and approved by the Institutional Review Board of Christian Health Association of Ghana (CHAG-IRB). If you have any questions about your rights as a research participant, you can contact the IRB Administrator, Mrs. Sarah Sackey Martei-Olletey on 0202904777 or email to [chagirb@chag.org.gh](mailto:chagirb@chag.org.gh).

**VOLUNTEER AGREEMENT**

The above document describing the benefits, risks, and procedures for the research title ……………………….............…………………………………... has been read and explained to me. I have been given an opportunity to have any questions about the research answered to my satisfaction. I agree to participate as a volunteer.

__________________________________________________________

Date, name and signature or mark of volunteer.

If volunteers cannot read the form themselves, a witness must sign here: I was present while the benefits, risks and procedures were read to the volunteer. All questions were answered, and the volunteer has agreed to take part in the research.

__________________________________________________________

Date, name and signature of witness.

I certify that the nature and purpose, the potential benefits, and possible risks associated with participating in this research have been explained to the above individual. _____________________________________________________

Date, name and signature of person who obtained consent

*As part of the consent process, participants were informed that their names and specific facility identifiers would not appear in any reports or publications, and that quotations would be presented using non-identifiable descriptors.*

## ***Supplemental Material 3***

*Code-book of the analyzed transcripts.*

| **Main Category (PNE)** | **Sub Category** | **Explanation** | **Anchor Example** |
| --- | --- | --- | --- |
| **Provider - Enabling Resources**  **Perceived Usefulness** | ICT Infrastructure | Refers to the availability and functionality of ICT tools such as network, electricity, hardware, and software. | “*I think [... in] Ghana, our network is not that good of quality. I think the main challenge will be the network. “*(Champion 14, Pos. 39) |
|  | Coordinated Services & Integration of Care | Refers to a unified healthcare approach where different departments work together. | *” We were having coordination. You have people from the lab, you have people from the pharmacy, you have doctors who were working with us and also the public health staff who were also working on the project. “*(Champion 15, Pos. 78) |
|  | Financial & Piloting Support | Refers to funding through external partnerships and pre-implementation piloting | *” I know that it was GIZ with Medtronic. And then I think Novartis. But I'm not too sure who were actually funding it. I know it's those three companies. Then they Liaised with CHAG. “*(Champion 20, Pos. 74) |
|  | Management Support & Commitment | Refers to the dedication and ongoing support from the management. | *” […] so, our medical director, he was very aware of everything and he encouraged us to do it. “*(Champion 4, Pos. 59) |
|  | Local Ownership & Integration in NGO-/ National Policy | Refers to the alignment of the intervention with national and NGO policies, as well as promoting local ownership. | *” I would want the government or major implementers to merge Akoma Pa with the national health insurance to help our patients. Yes, that would be very good for us. “*(Champion 6, Pos. 151)  *“And I think that, […] the funding, not some foreign people funding, it means the Kwame Nkrumah’s vision where he said that we are reads. We should be prepared to take care of ourselves, now is white people taking care of ourselves. [It] is more or less like a slavery or […] colonial powers […].” (Champion 3, Pos, 160)* |
|  | Available Physical and Technical Resources | Refers to the availability of physical and technical resources. | *“The computer, the BP machine, they gave us a phone and tablet.”* (Champion 14) |
|  | Evidence Based | Refers to evidence-based practices and data saved in the SPICE App. | *“You know, before Akoma Pa, there wasn't any valid data on these patients. So, we came into kind of fetch or put in place an accurate data on patients and how they do on medication.”* (Champion 2, Pos. 5) |
| **Provider - Enabling Resources**  **Perceived Ease of Use** | Data Management & Access | Refers to the enhanced data management through the SPICE app and the limited access to the data on the app. | *” So, the program really shows us how the patient is doing, and even, let's say, the blood pressure or the glucose levels. So, there is this graph that we normally see on the tablet that shows if the patient is progressing or de-progressing, or something like that.“* (Champion 10, Pos. 16) |
|  | User-Friendly Interface | Refers to the intuitive design of the intervention’s digital interface. | *” So, for the SPICE app, it was really easy to understand.“* (Champion 5, Pos. 33) |
|  | Maintenance & Technical Support | Refers to the ongoing technical support and maintenance of the equipment stated to be crucial for sustainability. | *” However, we have personnel who if the device goes wrong, they can repair it for us. “*(Champion 16, Pos. 35) |
|  | Extensive Interface & Interoperability | Refers to the suggestions of some further improvements to enhance interoperability to ease the work and communication between devices and departments and save up time used for double entries. | *“[…] if it is incorporated with the systems we are using currently, we wouldn’t feel it is giving us double work, because it is just the same thing you are doing. So if Akoma Pa needs to measure somebody's height, you just measure and put in. which is equally important for the LHIMS too”.* (Champion 23, Pos. 92) |
| **Provider – Needs**  **Perceived Usefulness** | Resource Gaps (Medical Supply, Human Resources & Financial Incentives) | Refers to the challenges of shortages of Medical Supply, Human Resources, and Financial Incentives. | “*Actually, when the drugs and the funding stopped, that is where the challenges started. “(Champion 1, Pos. 67)* |
|  | Workload Burden | Refers to the dual responsibilities of Akoma Pa and existing systems increasing workload through double entry. | *“Initially, what we were doing was that you see the clients on the LHIMS [ Lightwave Health Information Management Software], which is our health information and health management system. Then, you see the patients on the SPICE app as well. […] So, it wasn't easy because we were spending a lot of time doing that. “(Champion 20, Pos. 38)* |
|  | Transparency of Data Protection | Refers to unclear communication on data usage and eligibility. | „*So that kind of eligibility criteria was not explained well to us and so that was a challenge. And the aspect of NHIS being a requisite of registration.* “(Champion 27, Pos. 51)  *“Because we don't know where they are taking the data, and all those kinds of issues, make it clear and everything for us, understand, yes.”* (Champion 4, Pos. 67) |
|  | Service Delivery | Refers to the improved Service Delivery and Output because of the intervention showing increased attendance in the Outpatient Department (OPD). | *“I will say Akoma Pa has helped our facility to get more clients and it has helped the Akoma Pa clients too immensely. “*(Champion 30, Pos. 109) |
|  | Fostering Expectations | Refers to a switch of provider`s perspectives towards their expectations after the active phase. Early adopters often demonstrated excitement. | *“Okay, so, on the whole, I think my expectation was that this program would be sustainable for our clients, but it appeared that it's still not as effective as it started. So, I'll give it 50%.“* (Champion 13, Pos. 55)  *“Initially, they [expectations] were fully met because how they explained the project, how the projection of the project was, it came. The drugs came in numbers. They expected enrollment for the drugs and everything was up to date.”* (Champion 5, Pos. 82) |
|  | Misuse & Misappropriation of Resources | Refers to an instance of device misuse, where equipment got lost or providers took equipment home for personal use. | *“[…] sometimes you have the phones that we give to people. Sometimes take it home, it’s unavailable and there are a whole lot of challenges.”* (Champion 3, Pos. 87) |
|  | Inappropriate Workplace Behavior | Refers to a reported harassment by male patients through telecommunication, describing it as a challenge for female providers involved in the intervention. | *“The second part is that some males were taking advantage of us, “once you call them once or twice, they save your number, even at 12pm in the night, they’ll be calling you, that they can’t sleep and a whole lot of things, so some become a burden to us. It gets to a point some would confess that they were crushing on us, that’s why they were doing that. Clients-nurse relationships should not exceed its limits. So that kind of crossing limits was also a challenge because some were married, and it was worrisome.”* (Champion 27, Pos. 95) |
| **Provider – Needs**  **Perceived Ease of Use** | Effective Digital Tools and Features | Refers to effective digital tools and features in order to ease the use of this intervention. | *“Akoma Pa is also a type of e-health. And I think, like I said earlier on, Akoma Pa is good because with the app that we are using, there is a lot of information on it. We can even chat with the clients on the app. So, it's very helpful. “(Champion 19, Pos. 18)* |
| **Provider – Predisposing Characteristics**  **Perceived Usefulness** | Staff Engagement & Commitment | Refers to the provider`s commitment. | *“But now, since they realized the funding had stopped, the commitment went away.” (Champion 22, Pos. 62)* |
|  | Attitude & Willingness | Refers to overall attitudes towards the intervention ranging from enthusiastic to skeptical. | *“So, even right from its inception, we were having this doubt about durability, continuation and staff.”* (Champion 2, Pos. 44) |
|  | Good Patient-Provider Relationship | Refers to good relationships and interactions between patients and providers supporting satisfaction of patients. | *“And then you know some of our old people and all that. When you call them, they feel very loved. […] my healthcare provider actually thinks of me. It's nice that you're calling me.” (Champion 20, Pos. 78* |
| **Provider - Predisposing Characteristics Perceived Ease of Use** | Digital Literacy & Education | Refers to varying levels of provider`s Digital Literacy and Education, affecting their ability to engage with the intervention. | “*One disadvantage was that some struggled to use the app.”* (Champion 13, Pos. 69) |
|  | Continuous Training, Monitoring & Supervision | Refers to the availability of Continuous Training, Monitoring & Supervision for securing the ability and quality of work. | *“We were trained to do it and […] we were pre-informed of any update so there was no challenge. There was prior information before every time.” (Champion 28, Pos. 41)* |
| **Patient - Enabling Resources Perceived Usefulness** | Convenience, Affordability & Availability of Services | Refers to the reduction of financial and logistic barriers by bringing services closer to patients and minimizing costs. | *” Okay, so during the program, these free medications were a plus for the program because we realized that the patients who weren't coming to the facilities were due to financial problems.”* (Champion 10, Pos. 82) |
|  | Adequate Infrastructure & Resources | Refers to the availability of adequate infrastructure and resources in the facilities to support the intervention. Patients were happy about the quality of provided drugs. | *” That is when you ask anybody about Akoma Pa, they'll tell you: Oh, Akoma Pa, your medications are good. “(Champion 21, Pos. 85)* |
|  | Person-Centeredness | Refers to the program’s emphasis on person-centered care as an enabler of patient satisfaction regarding their expectations. A shift was seen from fulfilled expectations to unfulfilled expectations. | *“Some were even happy that the tele-nurses were calling them, informing them of their next schedules. So those expectations were actually achieved or met.”* (Champion 18, Pos. 65) |
| **Patient – Enabling Resources Perceived Ease of Use** | Family & Community Support | Refers to the support from family and community members, enhancing patients’ ease of engagement with the intervention regarding their access. Family members encouraged patients to attend appointments and assisted with using mobile devices. | *“When patients do not have phone numbers, we use our phone number and they bring a relative's phone number on the next visit, so that we can reach them later. “*(Champion 29, Pos. 32) |
|  | Health Insurance | Refers to health insurance facilitating the access and cost coverage for patients. | *“[…] the program was fully funded when it came in but now that the sponsorship isn’t there, we are now relying on the insurance for that. So the non-insured ones will have to make some payment.“* (Champion 23, Pos. 94) |
|  | Means of Identification | Refers to reliable means of identification needed to access/ register for the intervention. | *” Then every patient that comes, you have to register the person, generate a code for the person, then ensure that whenever the individual is coming, the patient will come with this card and then the insurance card as well.“* (Champion 2, Pos. 15) |
| **Patient – Needs**  **Perceived Usefulness** | Financial Burden of Medical Costs | Refers to the cost of medications being a barrier, with patients struggling to adhere to the program. | *“Now when they come, they pay for their drugs, so that is the challenge we have now.”* (Champion 28, Pos. 51)  *„So, even if we can't use the same Novartis or adapt that same medication, it should be a medication that all patients will be able to access or will be able to buy.“* (Champion 10, Pos. 47) |
|  | Awareness Raising | Refers to the importance of raising awareness about the intervention and its benefits. | *“The screening, we were moving from churches to churches, communities to communities, and the health education also went very well. So our people were able to concern with the initiative.”* (Champion 18, Pos. 41) |
|  | Polypharmacy Challenges | Refers to challenges related to polypharmacy, including medication side effects and adverse reactions. | *„I can count about three clients who came and told me that as they take the medication, they have palpitation. So, they wouldn't take it again. Though the BP came down, they said they were having the side effect of palpitation.“* (Champion 12, Pos. 65) |
|  | Access Barriers | Refers to patients in rural areas facing challenges related to distance, transportation, cost and time required to access services. | *“So, transportation to sites of screening and other stuff were a problem.“* (Champion 6, Pos. 67) |
|  | Management of Defaulters | Refers to the improved management and decrease of defaulters due to Akoma Pa. | *“But with this Spice app, we were able to track our patients. Those that were defaulting, we were able to see them and track them and call them back into the system. So, it really helped. “(Champion 6, Pos. 47)* |
|  | Improved Healthcare & Reduction of Disease Burden | Refers to improved health outcomes, better disease management and reduced burden of disease. | *“So, I can say it has saved a lot of lives. And also prevented some complications and disabilities as a result of hypertension and diabetes.”* (Champion 1, Pos. 8) |
|  | Information Accuracy | Refers to information provided by healthcare providers and the app which was mostly seen as beneficial. | *“So, information accuracy, what happens is that if we are managing a patient and we realize that the patient needs to know something about his or her conditions, we don't hesitate. We go ahead and we talk to them.“* (Champion 6, Pos. 58) |
| **Patient -  Needs Perceived Ease of Use** | Waiting Time | Refers to long waiting times at the healthcare facilities being a barrier to patients’ Ease of Use. | *“Because of the Akoma pa, we do the vitals 3 times (BP), and it delays the patient from going to see the doctor. We measure their height, weight and others. And measure their BP three times. So those who were to be part of the Akoma Pa would be shouting and complaining that we are wasting their time and delaying. So sometimes we have to skip it.“* (Champion 29, Pos. 37) |
|  | Tele-Counselling-Support | Refers to Tele-Counselling facilitating patient engagement and ease of use. | *“Later on, the Akoma Pa program was already doing the tele-counselling. So, we took it upon ourselves that sometimes we call them, and then we inform them about their visits, their next visits, and inquire from them how they are doing in their various zones.”* (Champion 10, Pos. 16) |
| **Patient – Predisposing Characteristics**  **Perceived Usefulness** | Health Literacy & Education | Refers to Health Literacy & Education which has shown to be helpful for patients` understanding of their condition. | *“And it has also helped patients who didn't know that they have such conditions. They've been able to know it and are able to get treatment.”* (Champion 8, Pos. 100) |
|  | Attitude | Refers to patients` Attitude to the intervention. | *“We've had people coming in very, very appreciative of the initiative.”* (Champion 5, Pos. 19) |
|  | Commitment & Attendance | Refers to patients` Commitment playing crucial roles in attending the intervention. | *“So, they realize the patient’s attendance increased.”* (Champion 22, Pos. 118) |
|  | Locality (Rural) | Refers to the geographical location in rural areas affecting the access to the mHealth intervention. | *“And also getting to where they are because we are from villages, trying to reach them was not easy. They coming to us was not easy. The distance was also a challenge.”* (Champion 12, Pos. 31) |
| **Patient – Predisposing Characteristics**  **Perceived Ease of Use** | Technological Access Barriers | Refers to Technological Access Barriers such as the lack of devices identified as barriers for implementation and scale-up. | *“[…] let me use my area for example. Some of them during our registration, you can't get their numbers because [in the] rural area.* Plenty of the ages, they don't use phones. And some of them who use phones don’t use the Android phones.” (Champion 15, Pos. 61) |
